# Interaction between rifampicin, amodiaquine and artemether in mice infected with chloroquine resistant *Plasmodium berghei*

**DOI:** 10.1186/1475-2875-13-299

**Published:** 2014-08-05

**Authors:** Joseph A Badejo, Oyindamola O Abiodun, Olugbenga Akinola, Christian T Happi, Akintunde Sowunmi, Grace O Gbotosho

**Affiliations:** 1 Department of Pharmacology and Therapeutics, College of Medicine, University of Ibadan, Ibadan, Nigeria; 2 Malaria Research Laboratories, Institute for Advanced Medical Research and Training, College of Medicine, University of Ibadan, Ibadan, Nigeria; 3 Department of Biological Sciences, Redeemer University, Mowe, Nigeria

**Keywords:** Malaria, Drug resistance, Amodiaquine, Rifampicin, *Plasmodium berghei*

## Abstract

**Background:**

Artemisinin-based combination therapy (ACT) remains the most effective chemotherapeutic strategy in the management of malaria. However, reports of reduced susceptibility of *Plasmodium falciparum* to the ACT justify the need for continued search for alternative anti-malarial drugs. The use of antibiotics with anti-malarial properties represents a potentially valuable chemotherapeutic option for the management of drug resistant infections. Thus, the intrinsic anti-malarial activity of the combination of clinical doses of rifampicin with amodiaquine and artemether was evaluated in an animal model using *Plasmodium berghei*.

**Methods:**

A modification of the suppressive tests *in vivo* was employed. The anti-malarial activity of standard doses of amodiaquine (AQ) with or without artemether (ART) and combined with varying doses of rifampicin (RIF 15 mg/kg or RIF 30 mg/kg body weight) was evaluated in 40 mice sub-divided into eight groups and inoculated intraperitoneally with 1 × 10^7^ red blood cells infected with chloroquine-resistant *P. berghei* ANKA strain. There were two control groups of animals, one group received amodiaquine alone while the other group received saline. Parasiticidal activity and survival of the animals were assessed over 21 days.

**Results:**

Parasitaemia in the control animals peaked at 38% on day 9 and all animals died by day 10. The combination of amodiaquine with rifampicin 15 mg/kg body weight was the most effective of all the combinations and more efficacious than amodiaquine alone. The order of superiority of anti-malarial efficacy of the combinations was as follows; AQ + RIF 15 > AQ > AQ + ART + RIF 30 > AQ + ART + RIF 15 > AQ + RIF 30.

**Conclusion:**

The combination of the clinical dose of rifampicin (15 mg/kg) with amodiaquine represents a potentially valuable treatment option in management of drug resistant malaria. In addition, the role of pharmacokinetic interaction in multiple drug therapy cannot be over-emphasized.

## Background

The World Health Organization recommends that artemisinin and its derivatives are combined with other anti-malarial drugs that have different mechanisms of action and longer half-lives in order to maximize the effectiveness of the artemisinins and to protect them from the development of resistance [1-3]. Artemisinin and its derivatives reduce most of the parasite biomass during their initial rapid action, while an effective partner drug can usually eliminate the small number of remaining parasites. In addition, the probability of emergence of a spontaneous mutation that confers resistance to two drugs with unrelated modes of action is very low [4]. Artemisinin-based combination therapy (ACT) remains the most effective treatment for uncomplicated *Plasmodium falciparum* malaria. Unfortunately, *Plasmodium* resistance to anti-malarial drugs continues to threaten effective control of malaria in endemic areas. Some evidence from *in vitro* monitoring over time in China and Viet Nam indicated increased IC-50, IC-90 or IC-99 values for artemisinins [5,6]. Furthermore, in a comparison of samples from Bangladesh, western Cambodia and western and eastern Thailand, decreasing *in vitro* susceptibility was observed from west to east [7]. In a separate study in Cambodia, the highest IC-50 was reported in the western part of the country [8]. Reduced susceptibility of *P. falciparum* to artemisinin derivatives in patients in western Cambodia has also been documented [9,10]. These reports hinder malaria control initiatives and since there are limited treatment options for the future, alternative treatment strategies are urgently needed.

The use of antibiotics with anti-malarial activities represents a valuable option in malaria chemotherapeutic strategies. Several antibiotics including tetracycline, azithromycin, fluoroquinolones and rifampicin have been shown to possess anti-malarial activity *in vitro* and *in vivo*[11-15]. Although most of the antibiotics are slow acting, they are best used in combination with a more rapidly acting drug [16-19]. In a study by Dahl and Rosenthal, clindamycin, ciprofloxacin, and azithromycin but not rifampin, caused delayed death in isolates of *P. falciparum in vitro*. The lower activity of the antibiotics has been attributed to a delayed effect of the drugs on the parasites, which becomes progressive with the duration of parasite-drug incubation [13,20].

Rifampicin is an antitubercular drug with potent anti-malarial activity against *Plasmodium vivax* in humans [21], *Plasmodium chabaudi* in rodents and chloroquine resistant *P. falciparum in vitro*[22]. Combination of rifampicin with isoniazid and co-trimoxazole was found to be effective in patients with *P. falciparum* infections [23]. Rifampicin appears to have a potential role to play in the management of malaria. However, multiple drug therapies increase the risk of pharmacokinetic drug-drug interactions which may be of clinical relevance. The availability of anti-malarial drugs over the counter (including the availability of artemether, amodiaquine or dihydroartemisinin tablets) especially in developing endemic countries promotes the concept of self-medication and this further confounds the challenges of drug-drug interaction. Rifampicin is a potent inducer of hepatic metabolism and can influence pharmacokinetics of other drugs [24] while artemisinin and its derivatives are metabolized by CYP 450 enzymes and can also induce the CYP enzymes [25,26]. Induction of metabolism is mediated mainly by the activation of the pregnane X receptor (PXR) and the constitutive androstane receptor (CAR) [27]. The artemisinin derivatives and their metabolites differentially affect the activities of CAR isoforms and of the pregnane X receptor [28] and this may have an impact on treatment outcome during multiple drug therapy. Efforts in this study were thus devoted to evaluating the interaction between rifampicin, amodiaquine and artemether *in vivo* during malaria infection in an animal model using *Plasmodium berghei.*

## Methods

### Drug samples

Rifampicin (RIF) was kindly provided by Bond Pharmaceuticals, Awe, Oyo State, Nigeria, and artemether and amodiaquine were obtained from the Walter Reed Army Institute for Research, USA. Stock solutions of the compounds were prepared in distilled water and stored at -20°C till required.

### Animals

The animals used in this study were male Swiss albino mice (6 – 8 weeks old) weighing 18 – 22 grams. The animals were obtained from the animal house of the Malaria Research Laboratories, Institute of Advanced Medical Research and Training (IMRAT), University of Ibadan, Ibadan. The mice were used in accordance with the NIH Guide for the care and use of laboratory animals, NIH publication (volume 25, number 28), revised 1996.

### Anti-malarial test *in vivo*

A modification of the suppressive tests in vivo [29] was used. Briefly, 40 male albino mice weighing 18 – 22 g were inoculated intraperitoneally with 1 × 10^7^ red blood cells infected with the CQ-resistant *P. berghei* ANKA strain. The infected animals were randomly divided into eight groups of five mice each and were treated by the oral route with rifampicin alone at 15 or 30 mg/kg body weight daily for seven days, amodiaquine 10 mg/kg body weight for three days + rifampicin 15 or 30 mg/kg daily for seven days or amodiaquine 10 mg/kg body weight for three days + artemether 8 mg/kg daily for three days + rifampicin or rifampicin 30 mg/kg body weight daily for seven days. Two controls groups were used, one treated with amodiaquine 10 mg/kg body weight given daily for three days while the second group of animals were treated with saline.

The stock solutions of the drug samples were diluted to the desired final concentration with distilled water so that each animal received 200 μl at time of administration of each drug. Parasiticidal activity was assessed daily from day 3 post-infection till day 14, and then on day 21. Blood smears were prepared from the tail, methanol-fixed, stained with Giemsa and microscopically examined by determining parasitaemia in 1,000 erythrocytes. Mortality was monitored daily, until four weeks after infection. Inhibition of parasite growth in drug-treated group was calculated in relation to parasite growth in the non-treated control group. All compounds and combinations were tested in three independent experiments.

### Statistical analysis

Student-*t* test was used to analyse the differences in mean parasitaemia level on days following treatment initiation, and analysis of variance between groups (ANOVA) was used to compare difference in percentage inhibition of parasite growth.

## Results

**Figure 1:**
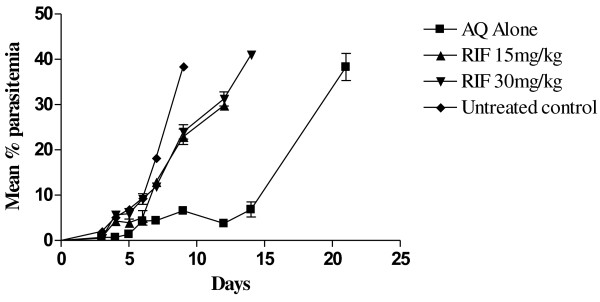
**Response of *Plasmodium berghei *** **infection in animals to amodiaquine or varying doses of rifampicin.**

Parasitaemia in the untreated control animals ranged from 2.0% on day 3 post-infection to 38.5% on day 9 when it peaked. All animals in the control group died by day 10. Parasitaemia did not clear completely in animals that received AQ or RIF alone. There was however, a significant decrease in parasitaemia (54% to 86%) in the animals treated with AQ alone during the follow up days (days 4-9) compared with the untreated control animals (P < 0.05) (Figure 1). In the group of animals that received RIF 15 mg/kg or 30 mg/kg alone, significant reduction in parasitemia (68% or 73% respectively) occurred only on day 3 post-infection compared to the control group. Parasitaemia was comparable to untreated control during subsequent follow-up days (P > 0.05). No mouse in the RIF 15 mg/kg group survived beyond day 12 post-inoculation while parasitaemia kept increasing in the RIF 30 mg/kg group until day 21 (parasitaemia = 68%) when the last mouse died (Table 1).

**Table 1:** **Survival of mice infected with chloroquine-resistant *P. berghei *** **(ANKA) and treated with amodiaquine alone or in combination with artemether and varying doses of rifampicin**

**Treatment Group**	**DAY 14**	**DAY 21**
AQ	40%	40%
RIF 15	0%	0%
RIF 30	20%	20%
AQ + RIF 15	100%	60%
AQ + RIF 30	60%	0%
AQ + ART + RIF 15	60%	0%
AQ + ART + RIF 30	60%	0%
Untreated control	0%	0%

### Comparison of intrinsic anti-malarial activity of AQ alone with AQ plus RIF

**Figure 2:**
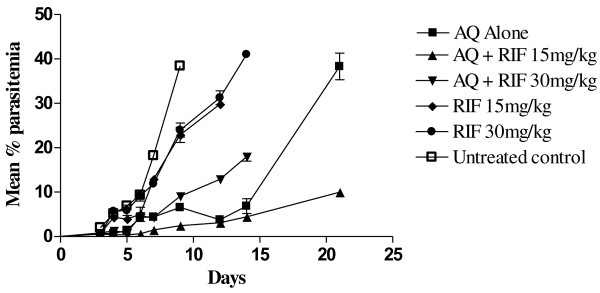
**Comparative response of *Plasmodium berghei *** **infections in mice to amodiaquine or rifampicin alone or amodiaquine in combination with varying doses of rifampicin.**

In the group of animals that received the combination of AQ plus RIF (15 mg/kg), mean parasitaemia was significantly lower (P <0.05) compared with the group of animals that received AQ only (Figure 2). In this treatment group, mean parasitaemia in the animals was 2.5 to 7 folds lower than mean parasitaemia in the group of animals that received AQ alone, between days 5 and 9 following treatment (Figure 2) and remained lower till day 21. Mean parasitaemia on day 9 (when parasitaemia in untreated control peaked at 38%) was lowest in the AQ plus RIF (15 mg/kg) treatment group and significantly lower than mean parasitaemia in AQ only group (2.44 ± 0.22% vs 6.57 ± 1.65% P = 0.034). Similarly, mean parasitaemia on day 21 in the animals that received AQ plus RIF (15 mg/kg) was approximately four times lower than mean parasitaemia in animals that received AQ alone (P < 0.05). In contrast, combination of AQ plus RIF (30 mg/kg) did not significantly lower parasitaemia in the animals rather response of infection in this treatment group of animals was comparable to response of infection in animals that received standard dose of AQ alone (P > 0.05) throughout the follow-up days (Figure 2).

### Comparison of intrinsic anti-malarial activity of AQ alone with AQ plus RIF and ART

**Figure 3:**
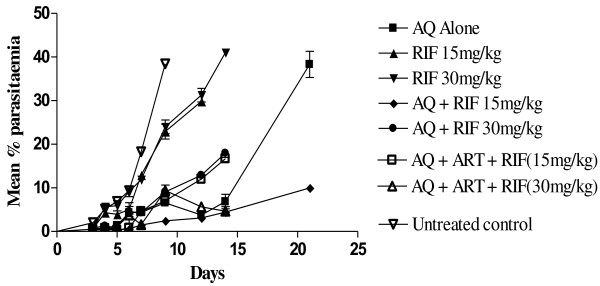
**Comparative response of *Plasmodium berghei *** **infections in mice to amodiaquine or rifampicin alone or amodiaquine in combination with varying doses of rifampicin and artemether.**

Parasitaemia in the group of animals treated with AQ + ART + RIF (15 mg/kg) or AQ + ART + RIF (30 mg/kg) significantly decreased by 75% to 96% or 76% to 94%, respectively, during follow up days 4 to 9 compared to the untreated controls (P < 0.05) (Figure 3). Mean parasitaemia on day 9 (when parasitaemia in untreated control peaked at 38%), in the group of animals that received AQ + ART + RIF (15 mg/kg or 30 mg/kg) was similar to mean parasitaemia in AQ only group (P > 0.05). Inclusion of ART in the combination of AQ + RIF (15 mg/kg or 30 mg/kg) did not significantly enhance response to treatment.

### Survival rate of animals

The survival rate of animals after treatment with AQ alone or in combination with varying doses of RIF and ART was assessed on days 14 and 21 and are presented on Table 1. The day 14 survival rate was highest (100%) in the group of animals that received AQ + RIF (15 mg/kg) while Day 14 survival rate was lowest in the group of animals that received AQ + RIF (30 mg/kg). Day 21 survival rate was 60% in the AQ + RIF (15 mg/kg) group, 40% in animals treated with AQ and 20% in the RIF 30 mg/kg group. No animal survived in the RIF 15 mg/kg, AQ + RIF (30 mg/kg) and ART + RIF (15 mg/kg or 30 mg/kg) treatment groups during the 21 day follow up.

## Discussion

Rifampicin a major component of combination treatment regimen for tuberculosis is derived from rifampicin B which is produced by *Streptomyces mediterranei*[24]. It is an antibiotic which has been shown to possess anti-malarial activity *in vivo* and *in vitro* in various experimental studies; Rifampicin is effective against *P. chabaudi* and *P. berghei* in rodents and chloroquine resistant *P. falciparum in vitro*[12,13,22,30]. Furthermore, beneficial interaction between rifampin and primaquine against *P. vivax* infection in humans has also been reported [21]. In that report, rifampin given at the usual dose of 15-20 mg/kg/day alone was active against the blood stage infection of human *P. vivax* malaria decreasing but not clearing parasitaemia.

This study describes beneficial interaction between amodiaquine and rifampicin in mice infected with chloroquine resistant strains of *P. berghei*. The interaction was demonstrated by a significant reduction in parasitaemia and enhanced survival rate of the animals (100% and 60% on day 14 and 21 respectively) in the group of animals treated with the combination of AQ plus 15 mg/kg body weight RIF. This is the first report describing beneficial interaction between RIF and AQ*.* Rifampicin alone given at standard clinical doses (15-30 mg/kg) in this study exhibited slight anti-malarial activity against *P. berghei*, as no substantial drop in parasitaemia was observed as reported in previous studies [22]. Strath *et al.* reported a clear dose-response effect of rifampicin with substantial drop in peripheral parasitaemia of *P. chabaudi* within 24 hrs of treatment following the use of 100-200 mg/kg body weight rifampicin. This dose is six folds higher than the dose employed in the present study. In a study by Schmidt, doses of rifampicin up to 20 mg/kg failed to effect cures or prevent relapses when combined with chloroquine [31].

The beneficial interaction between amodiaquine and rifampicin observed in this study especially at doses of rifampicin used clinically in the management of tuberculosis is a welcome discovery as co-existence between TB and malaria leads to various concerns about their treatment. The suitability of rifampicin rather than tetracycline for the treatment of children might be of particular interest in the potential clinical use of the combination. The combination of amodiaquine with 15 mg/kg bodyweight of rifampicin in this study was superior to the combination with 30 mg/kg bodyweight of rifampicin in reducing peripheral parasitaemia and enhancing survival rate. The reason for this disparity is not clear but it may not be unconnected with the auto-inducing effects of rifampicin which may also be dose related. Rifampicin is a great CYP450 inducer and one of the most potent inducers of all drugs used clinically [24]. Thus a decreased drug plasma concentration may result as rifampicin stimulates its own metabolism to inactive metabolites.

It would have been expected that the inclusion of artemether, a rapidly acting anti-malarial drug in the combination would produce a more significant effect in terms of enhanced parasite clearance and survival rate. Surprisingly, presence of artemether in the combination of amodiaquine with rifampicin did not produce a more superior anti-malarial effect over AQ + RIF 15 or AQ alone against the parasites. Superiority of the combination was as follows AQ + RIF 15 > AQ > AQ + ART + RIF30 > AQ + ART + RIF15 > AQ + RIF 30. The potential for drug-drug interaction cannot be overemphasized during combination or multiple drug therapy as it may pose serious challenges to treatment outcome. The lack of the expected enhanced anti-malarial effects when artemether was included in the combination may be as a result of pharmacokinetic interaction. Rifampicin is a potent inducer of hepatic and intestinal CYP450 enzyme activity (CYP3A4, CYP2C9, CYP2C8, CYP2C18/19) and all artemisinin derivative compounds are metabolized by the CYP450 enzymes to the active dihydroartemisinin, also available as a drug itself [25]. The artemisinins also seem to induce CYP 3A4 and CYP2C19 activity and their own metabolism [25,26]. Similarly, rifampicin is an auto-inducer stimulating its own metabolism into inactive metabolites [32]. Thus, concomitant administration of rifampicin with artemether may lead to an extensive time-dependent decline in drug plasma concentrations and a potential decrease in efficacy as a result of auto-inducing interplay between both drugs. This explanation is corroborated by a report by Lamorde *et al.* who reported unfavourable pharmacokinetic interaction between artemether, lumefantrine and rifampicin in healthy adults [33]. In that study, the area under the concentration-time curve (AUC) of artemether and dihydroartemisinin, which is a measure of exposure to drug was decreased by 89% during rifampicin treatment. In addition, the maximum plasma concentration of artemether and dihydroartemisinin were reduced by 83% in the presence of rifampicin. Bioavailability is concerned with how quickly and how much of a drug appears in the blood after a specific dose is administered and it often determines therapeutic efficacy since it affects the onset, intensity and duration of therapeutic response of the drug. Thus reduced plasma concentration and bioavailability of artemether in the presence of rifampicin may have contributed to the inability of the combination of amodiaquine/rifampicin/artemether to produce the expected enhanced effects against *P. berghei* in the present study. Similarly, reports of pharmacokinetic interaction between rifampicin and quinine which adversely affects the efficacy of quinine in uncomplicated falciparum malaria have been documented [34]. Pharmacokinetic interaction between rifampicin and quinidine or mefloquine have also been reported resulting in increased oral clearance of the anti-malarial drugs [35,36].

Theoretically it would be expected that rifampicin would alter amodiaquine activity when co-administered because amodiaquine is metabolized by CYP2C8, an enzyme also inducible by rifampicin. However this was not the case, as amodiaquine activity was significantly enhanced in the presence of rifampicin 15 mg/kg by enhancing the rate of fall in parasitaemia and survival rate of the animals. The higher dose rifampicin (30 mg/kg) did not appear to induce metabolism of AQ either, as response of infection to AQ + RIF30 was similar to that of AQ alone. Rifampicin auto induction, which seems to be dose dependent appears to be a plausible explanation.

In conclusion, this study reveals a beneficial effect of rifampicin in combination with amodiaquine against a chloroquine-resistant strain of *P. berghei* and provides a potentially useful chemotherapeutic alternative in the management of malaria in endemic areas. Detailed pharmacokinetic and toxicological studies on the interaction between amodiaquine and rifampicin are required prior to clinical application of the combination.

## Competing interests

The authors declare that they have no competing interests.

## Authors’ contributions

JAB participated in developing research protocol, data collection, data analysis and manuscript preparation, OOA participated in protocol development, data analysis, and manuscript writing, OA participated in data analysis and manuscript preparation, CTH participated in study design, protocol development, and data interpretation, AS participated in study design. GOG was responsible for conceptualization of study, study design, data analysis and interpretation, and manuscript preparation. All authors read and approved the final manuscript.
